# Psychosocial Experiences of Adolescent Girls and Young Women Subsequent to an Abortion in Sub-saharan Africa and Globally: A Systematic Review

**DOI:** 10.3389/frph.2021.638013

**Published:** 2021-05-19

**Authors:** Yasaman Zia, Nelly Mugo, Kenneth Ngure, Josephine Odoyo, Edinah Casmir, Eric Ayiera, Elizabeth Bukusi, Renee Heffron

**Affiliations:** ^1^Department of Global Health, University of Washington, Seattle, WA, United States; ^2^Department of Epidemiology, University of Washington, Seattle, WA, United States; ^3^Center for Clinical Research, Kenya Medical Research Institute, Nairobi, Kenya; ^4^Department of Community Health, Jomo Kenyatta University of Agriculture and Technology, Nairobi, Kenya; ^5^Center for Microbiology Research, Kenya Medical Research Institute, Nairobi, Kenya; ^6^Marie Stopes Kenya, Nairobi, Kenya; ^7^Department of Obstetrics and Gynecology, University of Washington, Seattle, WA, United States

**Keywords:** abortion, young women, adolescent and youth, sub-Saharan Africa, psychosocial, mental health, empowerment, global health

## Abstract

**Introduction:** Unmet need for contraception, unintended pregnancy, and access to safe abortion remain global challenges preventing adolescent girls and young women (AGYW) from achieving optimal reproductive health. Furthermore, AGYW face difficulties navigating sexual autonomy, lack of agency or experience negotiating sexual acts, and challenges accessing sexual health information and services. The aim of this systematic review is to assess the psychosocial outcomes of AGYW who have experienced an abortion with particular focus on sub-Saharan Africa, which bears the global burden of unintended pregnancy and risk of death due to unsafe abortion.

**Materials and Methods:** The systematic review was registered and used search terms to identify peer-reviewed articles relevant to “post-abortion,” “psychosocial,” “adolescent girls,” and “young women” from PubMed, Embase, PsycInfo, and Cumulative Index to Nursing and Allied Health Literature. Examples of psychosocial experiences include quality of life, stigma, and mental health outcomes. Rayyan software (Qatar, 2020) was used by two reviewers to assess the relevance of each article to psychosocial outcomes of AGYW any time after an abortion or accessing post-abortion services. Analysis was conducted with a focus on data from Africa and comparisons are made to non-African settings.

**Results:** A total of 2,406 articles were identified and 38 articles fit the criteria. Six selected articles were from Africa, including Ghana, Kenya, Uganda, and Zambia, and the remaining articles were from other regions. Themes around stigma, shame, and abandonment associated with the experience of abortion were prevalent in all regions. Studies of psychosocial outcomes of AGYW in sub-Saharan Africa highlight social isolation as well as learned resilience among young women who abort.

**Discussion:** Navigating abortion as an AGYW involves managing internalized and perceived stigma, fear of violence, secrecy, and growing resilient in order to overcome the significant barriers that society and culture place on access to an essential service in sexual and reproductive health. Post-abortion psychosocial outcomes highlight the need for support services and investigation of contexts that perpetuate and necessitate unsafe abortion. Empowerment of AGYW may present an important opportunity to build self-agency and positive coping mechanisms to withstand social pressures during stigmatizing circumstances associated with abortion.

## Introduction

Adults and adolescents alike possess the reproductive rights to have choice around when and how to parent (1). Yet unmet need for contraception, unintended pregnancy, and access to safe abortion remain global challenges preventing women, especially adolescent girls and young women (AGYW), from achieving optimal reproductive health (2). Globally, an estimated 121 million unintended pregnancies occur each year among women of reproductive age, and ~60% end in abortion (3). The rates of unintended pregnancy in sub-Saharan Africa are the highest worldwide, with ~91 pregnancies per 1,000 women aged 15–49 (4). Adolescents are particularly vulnerable to unsafe abortion because of the state of their cognitive development and the myriad of individual, interpersonal, and contextual influences that impact both their sexual health knowledge and behaviors and access to reproductive health services, including contraception (5–9). During adolescence and young adulthood, risk perception, and risk taking are contested against long-term consequences until the time when cognitive abilities mature through a neurodevelopmental growth period (10–12). AGYW face difficult challenges navigating their sexual autonomy given age-specific vulnerabilities, a lack of self-agency or experience in negotiating sexual acts, and difficulty accessing sexual health information and services (7, 13–15). On a social level, AGWY experience heightened awareness of social stigma pertaining to gender norms and their societal status as well as economic disempowerment sometimes leading to transactional sex (16, 17). Additionally, sexual and reproductive health information and services may be difficult to access due to cultural and social norms around adolescent sexuality, which creates stigma to accessing family planning services as well as safe abortion services and post-abortion care where available (18, 19).

Abortion is one of the pillars included in international normative guidelines for family planning, however 45% of all abortions occur in unsafe conditions with untrained providers and/or in facilities that do not meet minimal medical standards (20–22). Globally, the risk of dying due to an unsafe abortion is highest in Africa; an estimate of three out of four induced abortions in Africa occur in unsafe conditions, yielding a rate of ~520 deaths per 100,000 unsafe abortions (20). Most African countries legally restrict abortion and perpetuate cultural and religious stigma surrounding abortion, which often create barriers to accessing safe abortion and lead women to have an unsafe abortion (23). Even where the legal environment may be permissive of induced abortion under certain circumstances, significant barriers and social stigma result in inaccessibility of safe abortion sites, such as requiring parental consent for adolescents, lack of funds to pay for an abortion, dearth of information on safe abortion providers, and delays in timely care that may increase abortion-related health complications (24, 25). Additionally, extensive delays and difficulties in navigating the legality of abortion in Africa and globally can lead AGYW to seek unsafe abortions or self-induced abortions.

The social and cultural norms influencing the decision to abort are wrought with stigma. A largely contested topic is how these external and internalized ideologies impact the psychosocial outcomes of persons who choose to abort. Psychosocial experiences include a variety of factors such as quality of life, stigma, and mental health outcomes (e.g., depression, anxiety, post-traumatic stress disorder, mood disorders, and adjustment disorders). Among adult women worldwide, the presence and intensity of various feelings of guilt, regret, relief, and happiness after an abortion decrease over time, suggesting trajectories of emotional processing and coping (26). The most common psychosocial experience women cite is relief after an abortion (26–28). The preponderance of data show that long-term mental health outcomes of women who have had an abortion do not significantly differ from women who have not had an abortion (29). Currently, there is a growing epidemic of unintended pregnancy and abortion among AGYW living in sub-Saharan Africa and a gap in knowledge about their psychosocial experiences with abortion and how these experiences compare with their age counterparts in other world regions (30, 31). Thus, the aim of this systematic review is to assess the psychosocial outcomes of AGYW in post-abortion settings, with a focus on AGYW living in Africa.

## Methods

### Search Strategy and Selection Criteria

The protocol was registered with PROSPERO in June 2020 (ID: CRD42020197999). The systematic review used search terms to identify peer-reviewed articles relevant to “post-abortion,” “psychosocial,” “adolescent girls,” and “young women” from the following search engines: PubMed, Embase, PsycInfo, and Cumulative Index to Nursing and Allied Health Literature (CINAHL) (full terms for each database are listed in the Appendix in Supplementary Material). A tracking reference strategy was used to ensure that articles that met the criteria were included in the search results. All articles identified from the four databases published prior to September 30, 2020, were included. Inclusion criteria included articles that captured psychosocial experiences at any point after an adolescent or young woman's abortion. Exclusion criteria included lacking data on AGYW under 25 years old (or unable to be disaggregated from older age groups), not related to induced abortion (e.g., related only to spontaneous abortions or fetal anomalies), focused on psychosocial experiences before or during an abortion, irrelevant data (provider focused, wrong population), being non-original publication (such as commentary, letters to the editor, news articles, policy briefs, and guidance documents), and lack of an English version of the abstract and/or the article being available. Articles were imported into Rayyan software (Qatar, 2020) and duplicates were excluded. Two reviewers (YZ and RH), who are both cis-gendered women from the United States, read all article titles and abstracts to assess the relevance of each article to the predetermined criteria, particularly that psychosocial outcomes were measured in post-abortion settings and that data pertaining to AGYW were included. Finally, the same two reviewers assessed the conflicts on initial review decisions and discussed discrepancies to reach agreement. All remaining articles were included for analysis.

### Analysis

The analysis was stratified by region with experiences from Africa being centered and data from other regions being utilized to compare and contrast. Data from articles were extracted to identify all themes related to mental health and grouped together to synthesize data across each theme. The data were extracted systematically by YZ through content analysis, focusing on the experiences described in the qualitative articles in Africa, to inform the initial themes that arose from the included articles. These themes were then discussed with RH and, through location-level thematic analysis, compared and contrasted to the findings in other regions to consolidate main themes. In addition to the predominant psychosocial themes identified in Africa, data from other regions were amalgamated to describe trends that were not captured within the African region.

## Results

**Figure 1:**
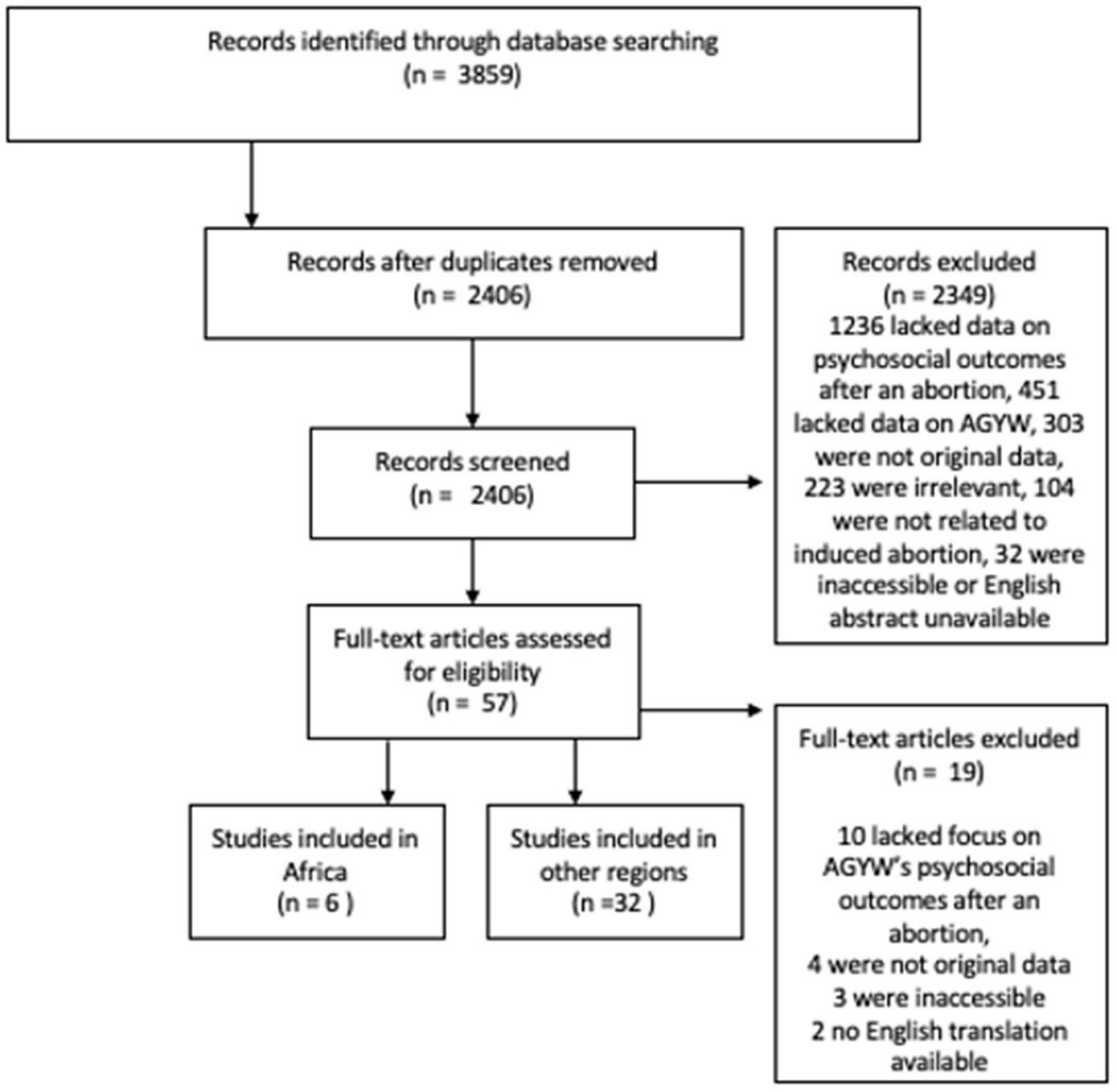
PRISMA flow diagram.

A total of 2,406 de-duplicated articles were identified. Based on review of titles and abstracts, 2,349 were excluded due to irrelevance based on population, outcome or exposure and/or lacking data about psychosocial outcomes after an abortion among AGYW, focusing on spontaneous abortion or abortion due to fetal anomaly, lacking original data, or missing English translation. After these exclusions, 57 articles met the criteria for full manuscript review. Upon in-depth review of these 57 articles, 10 were excluded because they lacked focus on psychosocial outcomes of AGYW after an abortion, 4 did not contain any original data, 3 were inaccessible, and 2 did not have an English translation of the manuscript. After the final exclusions, 38 articles remained for analysis of the psychosocial outcomes of AGYW following an experience with abortion (PRISMA Figure 1). There were six articles that originated from Africa and 32 articles that were from other regions.

### Overview

The selected articles from Africa were quantitative and qualitative studies from Ghana (*n* = 2), Kenya (*n* = 2), Uganda (*n* = 1), and Zambia (*n* = 1, Table 1). There were 32 quantitative and qualitative articles included outside of Africa, with studies from Asia (India), Europe (Bosnia and Herzegovina, England, Finland, Norway, Sweden), Central and South America (Brazil, Chile, Mexico), Oceania (Australia, New Zealand), and North America (United States). After an abortion, the experiences related to psychosocial outcomes that AGYW most predominantly expressed included social isolation and abandonment from family, friends, and partners; stigma perpetuated through legal, social, and religious messaging; and self-reliance and increased agency. Some AGYW reported that their abortion was “the most challenging life-decision ever” (35). AGYW expressed a range of reactions to and emotions surrounding their abortion including confusion, shame, guilt, and fear, but also self-agency and empowerment. Outside of Africa, the context through which an AGYW received an abortion informed their psychosocial outcomes, including parental and partner support, timely access to services, and cultural and internalized stigma around abortion (38–44).

**Table 1:** Summary of articles of psychosocial outcomes of adolescent girls and young women in Africa.

**Study**	**Year published**	**Study design**	**Location**	**Study goal**	**Key findings**	**Unique findings**	**Themes**
**Summary of quantitative analyses**								
Mutungi et al. (32)	1999	Cross sectional survey among school adolescents (35/1,024 girls and 52/558 boys who reported an abortion) and 192 post-abortion recipients	Kenya	To evaluate the adolescents' behavior regarding induced abortion.	• Direct and indirect costs of abortion were heavy on the girls, including financial costs (>1,000 Kenyan shillings) • 70% report losing time at school • The prevalence of self-reported abortion among school adolescents was ~5.5%.	Approval of abortion was <50% among school adolescents who received an abortion and almost 80% among those in a PAC setting.	Internalized Stigma, Abandonment, Gender Violence, Secrecy, Empowerment
**Summary of qualitative analyses**								
Mohamed et al. (33)	2018	IDI with 15 AGYW	Kenya	To characterize Kenyan women's perceptions and experiences with abortion and post-abortion care (PAC) services in Nairobi regarding barriers to care, beliefs about abortion, and perceived stigma.	• Perceived stigma was the most significant psychosocial barrier respondents faced in promptly seeking abortion and PAC. • In response to abortion, women developed a sense of agency and self-reliance and learned to prioritize their own healthcare needs over the concerns of others.	Despite the heavy presence of abortion stigma, most participants expressed agency over their decision. Even those who felt strongly that abortion is wrong, still expressed feeling that they made the right decision.	External Stigma, Punishment, Secrecy, Resilience
Aziato et al. (34)	2016	Vignette-based FGD with 92 AGYW	Ghana	To investigate the experiences and perceptions of adolescents who have experienced a recent pregnancy and undergone a termination of pregnancy through a vignette of an adolescent who has recently become pregnant.	• Describe feeling sadness, depression, and regret from an unintended pregnancy and that some male partners would “deny” the pregnancy or suggest abortion. • Parents might send the pregnant girl to a distant friend or grandparents until she delivers to avoid shame and gossip. • Most health professionals would insult/gossip about the girl.	Decisions around adolescent unintended pregnancy is often shaped through partners, partners, and health professionals.	External Stigma, Punishment, Secrecy, Resilience
Esia-Donkoh et al. (35)	2015	IDI with 21 AGYW	Ghana	To examine the pre- and post-abortion experiences of young females.	• Fear of societal stigma, shame, and rejection by partners. • Self-imposed stigma reinforced by social and religious messages.	AGYW were worried about “illegality,” “crime,” and sin had been committed against humanity and God leading to self-imposed stigma, which was reinforced by sermons and talks about abortion at the Church.	Internalized Stigma, Abandonment, Secrecy, Resilience
Cleeve et al. (36)	2017	IDI with 17 AGYW	Uganda	To explore reproductive agency in relation to unsafe abortion among young women seeking post-abortion care.	• Reproductive agency was constrained by gender norms and power imbalances and strongly influenced by stigma. • Lack of resources and the need for secrecy resulted in harmful abortion practices and delayed care-seeking. • Abortion as an agentive action aiming to regain control over one's body and future.	Women did not claim ownership of the abortion decision, and still abortion represents a vital form of female empowerment.	External Stigma, Punishment, Gender Violence, Secrecy, Resilience
Dahlbäck et al. (37)	2010	Mixed methods, Semi-Structured Questionnaire with 87 AGYW after spontaneous pregnancy loss or clandestine abortion	Zambia	The aim was to explore experiences of pregnancy loss and to ascertain the girl's contraceptive knowledge and use and their partner's involvement in the pregnancy/abortion.	• Sexual coercion was common (34% in spontaneous abortion and 44% in clandestine abortion group). • Becoming an unmarried teenaged mother abandoned by the partner was considered stigmatizing, humiliating and a shame for the girl herself and for her family. • Choosing between having an unsafe induced abortion versus giving birth and being emotionally relieved vs. feelings of guilt and sin was frequently mentioned.	None of the girls in the study said they were aware of the current abortion law in Zambia and the possibility of having a legal abortion.	Internalized Stigma, Abandonment, Gender Violence, Secrecy, Resilience

*AGYW, Adolescent girls and young women; IDI, In-depth interview; FGD, Focus group discussion*.

### Internalized and Perceived Stigma

Throughout the articles from Africa, a predominant qualitative theme was the trickling down of societal norms, resulting in AGYW experiencing internalized and perceived stigma following abortion. Perceived stigma refers to external social factors such as opinions of parents or healthcare providers, whereas internalized stigma is the development of negative feelings toward oneself and acceptance of stereotypes or prejudice (45). For example, in one study of 87 AGYW in Zambia, internalized stigma and shame led AGYW to weigh the consequences of abortion vs. continuing an unwanted pregnancy and the feelings of emotional relief vs. guilt and sin associated with abortion (37). In another study of in-depth interviews (IDI) with 21 AGYW in Ghana, self-imposed stigma and belief that abortion was a sin was reinforced by the illegality of abortion, religious sermons, and media and other social platforms (35). Even among AGYW who had an abortion, some (30–60%) still indicated a continued view of abortion as unacceptable or did not claim ownership of their decision indicating internalized stigma (32, 37).

In addition to their individual experiences, AGYW in Africa heavily deliberated how their abortion would be viewed by others (34). Prevalence of stigma and shame extending from the AGYW to family regarding premarital sex, teenage pregnancy, and abortion were often cited (33, 34, 36). Additionally, social exclusion and abandonment were predominant deleterious themes surrounding an AGYW's abortion in African settings. Data from Ghana describe how some families send their pregnant daughters to stay with extended family members or other members of their social network in other villages to avoid stigma within their local setting (34). Partnership dissolution, including abandonment, was also a major concern for AGYW in Zambia and Ghana when revealing their abortion (35, 37). AGYW described the difficulty of needing emotional support after their abortion and being physically and emotionally ostracized by their family and partners as a result of their decision to abort (35, 37). Healthcare providers and broader community shaming or gossiping about the AGYW's abortion were also mentioned in terms of external stigma (34). In summary, abortion stigma is pervasive globally and causes psychological distress in AGYW through anti-abortion attitudes held by parents and partners and perpetuated through healthcare systems and religion and legal regulations (46–49).

### Violence and Gender Dynamics

Themes around physical punishment or gender-based violence often influenced AGYW decisions to abort and their subsequent well-being. In multiple studies in Africa, AGYW expressed feeling threatened to be killed or beaten, by either their parents or partners, for their decision to abort (33, 34, 36). Studies outside of Africa also showed that intimate partner violence was a significant contributor to depression experienced by AGYW after abortion (50). In addition to threats of physical violence, women experiencing abortion describe challenges with gender dynamics and power imbalances. Gender-based violence around sexual decision-making impact psychosocial well-being of AGYW. Sexual coercion to dissuade condom use or timing of condomless sex are cited among AGYW in Africa (36, 37). Financial dependence and transactional sex also reinforce the gender norms that women are economically reliant on men, and therefore less able to negotiate sexual activities (36). These power imbalances constrain AGYW's reproductive agency and contributed to the context in which the unwanted pregnancy occurred.

### Secrecy and Social Isolation

Many women described desire to keep the experience of their abortion secret in order to avoid stigma, shame, and/or violence and also recognized the way that secrecy contributed to social isolation and increased health risks during and after an abortion. In Zambia, despite the legality of abortion, stigma and secrecy sometimes led to unsafe abortion or self-induced abortion (37). Further, keeping an abortion a secret increased health risks in seeking care from untrained providers who often operate in clandestine spaces at poorly equipped facilities or withholding information from healthcare providers (33, 36). AGYW described acting ignorantly about sexuality and family planning to avoid being viewed as promiscuous or sexually active, and therefore purposefully avoid seeking sexual and reproductive health knowledge and services (37). Dissuasion from family planning and secrecy around sexual health needs also result in repeated abortions, which reinforce the continued inaccessibility of information and services for AGYW to prevent future unintended pregnancies (32). Secrecy also led AGYW to go through the abortion experience alone and often without proper counseling or emotional support. Social isolation reinforces the cultural and social shame and projects these ideologies onto AGYW who become pregnant as a way to maintain traditions around gender norms (33). However, their secrecy about their abortion may have also provided agency to maintain their decision-making power and practice their right to bodily autonomy (36). Secrecy was a theme globally as well, with various levels of maintaining secrecy after an abortion from parents or partners (38). Overall, AGYW are willing to experience vulnerability physically, socially, and emotionally by keeping their abortions an autonomous decision.

### Resilience

In contrast to stigma and violence, some studies recognized that the experience of self-reliance and self-determination to abort a pregnancy provides AGYW with a sense of empowerment and agency in prioritizing their healthcare needs (33). Despite the restrictive legal contexts and pervasive cultural and religious stigma, AGYW in Kenya and Uganda studies described their abortion perceptions and related psychosocial outcomes as empowered (33, 36). Facing stigma whether carrying to term an unwanted pregnancy or terminating the pregnancy through abortion, AGYW continued risking their lives and reputations for the autonomy of choice (36). The incompatibility of adolescent pregnancy with AGYW's schooling and professional pursuits, fiscal requirements and subsequent costs to have a child, and lack of social support presents a pragmatic reality through which adolescents in two studies rationalized the decision to abort (34, 36). The predominant theme of AGYW rationalizing an abortion was centered on the idea that while abortion was thought of as immoral, illegal, or unacceptable, it was the only option that would permit AGYW to continue seeking their future goals with schooling and professional pursuits (35, 37). Empowerment was cited from studies taking place outside of Africa as well (39, 40), with themes around increased self-esteem and self-image and higher chances of continuing education or staying in school expressed by AGYW experiencing an abortion in Brazil and the United States.

### Themes Captured in Other Regions

Themes of psychosocial factors that were captured in studies outside of Africa that were not measured in Africa include depression, post-traumatic stress disorder, drug and alcohol use, sexual well-being, and other mental health outcomes (Table 2). In terms of mental health outcomes after an abortion, data on whether abortion impacts mental health outcomes among AGYW are inconclusive as some data suggest worse outcomes among those who choose to abort (50–54, 56, 57) while others indicate no increased risk of depression, suicide, or post-traumatic stress disorder associated with abortion (55, 58–66). However, there are important limitations in these 17 studies that aim to determine associations between mental health outcomes and the abortion experience including that the comparison groups across these studies range from the same AGYW pre-abortion, non-pregnant or sexually inactive AGYW, AGYW who chose to continue pregnancy, or women who aborted later in life, reducing the comparability of these studies to one another. In these studies, factors that influenced mental health outcomes included partner violence, internalized stigma, wantedness of the pregnancy, and contextual factors such as education, income, and family support (38, 50, 57, 63). Additionally, four studies assessed levels of alcohol, nicotine, drug use, and dependence among AGYW who had an abortion. Smoking, drug use, and alcohol use were increased under certain circumstances among AGYW who had an abortion (50, 67–69), although behaviors prior to an abortion and the continued experimentation in the neurodevelopmental phase of adolescence and early adulthood may be motivating these findings. Lastly, sexual well-being was assessed in two studies and indicated that AGYW had high levels of sexual satisfaction and function (61, 70). Overall, the studies outside of Africa, specifically those in Europe, Oceania, and the United States, tended to be survey or registry-based studies of psychosocial outcomes and identified additional themes around mental health diagnoses and frequency of above-mentioned behaviors that were not captured in Africa.

**Table 2:** Summary of psychosocial outcomes of adolescent girls and young women in other global regions, by key theme.

**Study**	**Year published**	**Study design**	**Region**	**Key finding**
**Theme: Increased depression, post-traumatic stress disorder, suicide**				
Dingle et al. (50)	2008	Retrospective Cohort	Australia	Abortion during adolescence was associated with current depression (OR = 1.9, 95% CI: 1.1–3.1) among 21-year-olds.
Zulčić Nakić et al. (51)	2012	Case-control	Bosnia	Adolescents who aborted pregnancy had significantly greater depression symptom severity and frequency than adolescents who did not abort.
Jalanko et al. (52)	2017	Cohort	Finland	The abortion group faced higher risks of suicide and dying from injury and poisoning compared with AGYW who had given birth.
Fergusson et al. (53)	2006	Prospective Cohort	New Zealand	Those having an abortion had elevated rates of subsequent mental health problems including depression, anxiety, suicidal behaviors, and substance use disorders.
Pedersen et al. (54)	2008	Prospective Cohort	Norway	Young women, but not teenage women, who reported having had an abortion in their twenties were more likely to score above the cut-off point for depression.
Ely et al. (55)	2010	Cross-sectional	United States	Forty percent of adolescent pregnancy termination patients reported elevated levels of depression, which was also closely related to stress, anxiety, and self-esteem issues.
Sullins (56)	2016	Prospective Cohort	United States	Risk of mental disorder is higher (1.62) for teenage women who have abortions, compared to AGYW over age 20 (1.51).
**Theme: Decreased or no effect on depression, post-traumatic stress disorder, suicide**				
Taft and Watson (57)	2008	Cohort	Australia	Thirty percent of AGYW reported depression, however violence, especially partner violence, makes a significantly greater contribution to women's depression compared with pregnancy termination or births.
Jalanko et al. (58)	2020	Cohort	Finland	Women who underwent an abortion at <18 years of age instead of childbirth faced a lower risk of psychiatric morbidity, particularly during the first 5 years postabortion.
Leppälahti et al. (59)	2016	Retrospective Cohort	Finland	No significant differences between the underage abortion and the childbirth group regarding risks of psychiatric disorders, as psychiatric disorders and risk-taking-related health outcomes, including injury, were increased in the abortion group and in the childbirth group similarly on both sides of the pregnancy.
Gomez (60)	2018	Prospective Cohort	United Kingdom	In a nationally representative, longitudinal dataset, there was no evidence that young women who had abortions were at increased risk of subsequent depressive symptoms compared with those who give birth after an unwanted first pregnancy.
Limoncin et al. (61)	2017	Prospective Cohort	United Kingdom	Women did not fall into clinically significant depression or anxiety before abortion, and their scores significantly decreased at 6 months post-abortion.
Pereira et al. (62)	2017	Cross-sectional	United Kingdom	Adolescents experiencing abortion are not at greater risk of psychosocial maladjustment than are adult women.
Pope et al. (63)	2001	Prospective Cohort	United States	There was no evidence that abortion poses a threat to adolescents' psychological well-being; AGYW under age 18 years were less comfortable with their decision, but showed no other differences compared with those aged 18–21 years.
Zabin et al. (64)	1989	Prospective Cohort	United States	An analysis of psychological stress showed that those who terminated their pregnancy had experience no greater levels of stress or anxiety than had the other teenagers at the time of the pregnancy test, and they were no more likely to have psychological problems 2 years later.
Warren et al. (65)	2010	Prospective Cohort	United States	Adolescents who have an abortion do not appear to be at elevated risk for depression or low self-esteem in the short term or up to 5 years after the abortion.
Schmiege and Russo (66)	2005	Prospective Cohort	United States	Terminating compared with delivering an unwanted first pregnancy was not directly related to risk of clinically significant depression.
**Theme: Drug and alcohol use**				
Olsson et al. (67)	2014	Prospective Cohort	Australia	Compared to AGYW who were never pregnant, those who terminated a pregnancy had a higher risk of smoking and alcohol use as well as nicotine and alcohol dependence
Dingle et al. (50)	2008	Retrospective Cohort	Australia	Women who had an abortion were twice as likely as women who were never pregnant or who gave birth to have an alcohol disorder.
Pedersen (68)	2007	Prospective Cohort	Norway	Those who had had an abortion had elevated rates of substance use and problems, except under circumstances where they lived with their partner.
Tung et al. (69)	2020	Prospective Cohort	United States	Abortion was not associated with long-term changes in substance use; however, marijuana and cigarette use gradually increased (44–46%) in the years leading up to the year of and after abortion, respectively, before returning to pre-pregnancy levels.
**Theme: Sexual well-being**				
Pohjoranta et al. (70)	2018	Clinical Trial	Finland	Sexual well-being does not change significantly after termination of pregnancy. Instead, it is strongly and positively associated with quality of life, relationship status and frequency of intercourse. Anxiety is negatively associated with sexual well-being.
Limoncin et al. (61)	2017	Prospective Cohort	United Kingdom	Adolescents had high mean total Female Sexual Function Index scores before an abortion and mean scores were increased 6 months after an abortion.
**Theme: Other qualitative work and general abortion themes**				
Bailey et al. (41)	2001	Cross-sectional	Brazil	Teens who terminated their pregnancies were the most likely to be in school or working 1 year later. Compared to AGYW who gave birth, they also showed the greatest increase in self-esteem.
Domingos et al. (44)	2013	Qualitative	Brazil	After the abortion demanded by the AGYW's mother, AGYW experienced suffering, guilt, and regret for not having fought against their mothers' decisions.
Palma Manríquez et al. (38)	2018	Qualitative	Chile	Clandestine abortions made AGYW physically, socially, and emotionally vulnerable and exposed them to the risk of normative, violent judgments during post-abortion care.
Jejeebhoy et al. (43)	2010	Mixed methods	India	Unmarried AGYW were also more likely to report obstacles to timely abortion as failure to recognize the pregnancy promptly, exclusion from abortion-related decision-making, seeking confidentiality as paramount in selection of abortion facility, unsuccessful previous attempts to terminate the pregnancy, and lack of partner support.
Sorhaindo et al. (48)	2014	Qualitative	Mexico	AGYW expressed doubt that Catholic Church's perspectives were fair to the reality of many women's lives, and there are other aspects to consider when a woman has an abortion (financial means, psychological capacity for motherhood, personal desires at school, work) not just moral.
Hoggart et al. (47)	2017	Qualitative	United Kingdom	Women who had experienced more than one abortion express intensified abortion shame.
Felton et al. (42)	1998	Cross-sectional	United States	No significant differences comparing health behaviors, problem-solving appraisal, self-image, age, and use of contraceptives at first and most recent coitus in adolescents with a history of abortion and never-pregnant adolescents.
Andrews et al. (39)	2003	Qualitative	United States	As African–American AGYW moved through the experiences of unplanned pregnancy and elective abortion, the participants gained control and refined their ability to make decisions.
Ralph et al. (40)	2014	Cross-sectional	United States	Most minors involved parents and partners in their decision making regarding abortion and find support from these individuals. For some, experiencing pressure or lack of support reduced confidence in their decision and increased their likelihood of anticipating poor coping after an abortion.
Gelman et al. (46)	2017	Qualitative	United States	Women reacted to external and internal negative attitudes by distinguishing themselves from other women who obtained abortions, experiencing negative emotions, and concealing or delaying their abortions.
Coleman-Minahan et al. (49)	2019	Qualitative	United States	In addition to unpredictability and logistic burdens such as finding time away from school and arranging transportation, AGYW < 18 described the required Texas judicial bypass process as “intimidating” and “scary” and described judges as people who shamed them, “preached” at them, and discredited evidence of their maturity.

*AGYW, Adolescent girls and young women*.

## Discussion

This review consolidates data globally, across 6 studies in Africa and 32 studies in other regions, that shape the experiences of AGYW who can access an abortion. Taking into consideration the lack of literature on AGYW after an abortion in Africa, this review centered on AGYW's psychosocial experiences in the African region and linked themes globally across other regions. Studies of psychosocial outcomes of AGYW in Africa highlight experiences with shame and isolation as well as learned resilience among young women who abort. Comparing the experiences of AGYW in Africa to other regions, themes around stigma, shame, gender dynamics, and abandonment were prevalent in all regions. Given the diversity of cultures and social contexts both within and outside of Africa, there exist significant underlying differences in psychosocial outcomes among AGYW in high vs. low income settings, countries where abortion is legal vs. restricted or illegal, and rural vs. urban settings; however, the themes of societal and internalized stigma, parental, and partner involvement or lack thereof, and obstacles in timely care were apparent globally (38–44, 46–49). The AGYW's abortion experience is contextualized through the prism of influence of her partner(s), parents, and providers, messages she receives through cultural and social norms, and her own resolve to make pragmatic choices for her life. Navigating abortion as an adolescent or young woman involves managing internalized and perceived stigma, fear of violence, secrecy, and growing resilient in order to overcome the significant barriers that society and culture place on access to an essential service in sexual and reproductive health.

The psychosocial outcomes experienced by AGYW after an abortion highlight the need for support services and better understanding of the contexts that perpetuate and necessitate unsafe abortion. While the included studies did not capture research on specific support services for AGYW, a range of education and support services from prevention through post-abortion can enhance psychosocial outcomes of AGYW. Gender dynamics in sexual negotiation are a societally propagated norm. As an aspect of prevention and sexual health promotion, education and services that promote condom and sexual self-efficacy among adolescents may improve circumstances surrounding sexual decision-making and consent (13–15, 71). Leading up to and immediately after an abortion, parental social support may improve post-abortion coping and accompaniment immediately after an abortion can improve anxiety (40, 72). However, not all AGYW have access to parental support, and the context of secrecy and stigma may pose barriers to disclosing an unintended pregnancy and/or experience with abortion to a parent. As an existing gap in the care continuum after an abortion, healthcare workers often do not follow up to check how AGYW are coping psychologically. Post-abortion counseling services can be used to evaluate the need for referral to a mental health specialist, going beyond the narrow focus on contraceptive counseling (73). In settings, where societal and social environment heavily influence stigma surrounding adolescent sexuality and may subsequently escalate psychosocial harm among AGYW seeking reproductive health services, access to support services for AGYW prior to sexual debut through post-abortion settings may allow AGYW to prevent unintended pregnancy and/or process the internalized shame after an abortion (19). Support for AGYW's reproductive health include comprehensive school-based sexual health education; a strengthened healthcare system that uses patient-centered, non-judgmental care for AGYW; and advocacy and social support disseminated across levels of mass media, community, and family (74).

Empowerment of AGYW in making the decision to abort and in post-abortion reflection may present an important opportunity to build self-agency and positive coping mechanisms within stigmatizing circumstances (33, 39, 40). No published studies in Africa utilized validated mental health screening tools to assess psychosocial outcomes among AGYW or to estimate the impact that becoming more empowered may have on AGYW accessing abortion services. Sexual and reproductive empowerment can be measured well with validated questionnaires or qualitative studies and data can be used to frame the abortion and post-abortion experiences of AGYW with resilience or agency (75). Validated, accurate, and culturally relevant measurement of these outcomes is essential in order to identify other themes. Given the stigma surrounding abortion and difficulty of sharing abortion stories, these tools for measuring mental health and sexual empowerment may be utilized to improve quality of data captured in study design and to address the gaps in knowledge of AGYW's experiences of abortion.

The review was limited in scope and focused on AGYW in Africa with articles from other settings used to compare and contrast; therefore, it does not describe all the psychosocial outcomes that can be experienced subsequent to abortion or the contexts in which abortion occurs. Key outcomes needing further review include experiences of abortion among people with mental and physical disability, people who identify as gay, lesbian, bisexual, pansexual, queer, transgender, and/or gender non-conforming, the influence of humanitarian crises on abortion decisions and experiences, and the weight of rape and sexual coercion on abortion decisions and experiences. Psychosocial outcomes attributable to sexual coercion in condomless sex or timing of sex through emotional pressure are difficult to untangle from those of abortion. We excluded data from young men whose partner had an abortion, and these men may experience psychosocial outcomes of their own. While our search terms attempted to cast the broadest net to capture any psychological and social factors experienced by AGYW after an abortion, it is possible that studies relevant to this topic were excluded if they were published after our search or potentially due to the mapping of our search terms or the judicious focus on AGYW < 25 years old. The bulk of the articles from other regions stemmed from the United States (*n* = 12), and data from certain regions of the world were missing, which was not due to a lack of English translations. The body of literature is missing data on psychosocial experiences specific to AGYW from East Asia, Middle East, and Central Asia. Additionally, the articles included from Africa are from four countries and do not represent all of Africa, and the experiences of AGYW in other African countries may vary from those presented here.

Since abortion is steeped in stigma and secrecy, there is likely underreporting of unsafe and clandestine abortions, and the mental health burden experienced by AGYW accessing (safe and unsafe) abortion remains poorly addressed and understudied, especially in Africa. Shame and stigma surround decisions to terminate a pregnancy, and these feelings contribute significantly to and create undue burdens on psychosocial experiences of AGYW. Abortion is a moralized and ideological dilemma reflected in both society and the AGYW navigating the abortion experience, and further research is needed on stigma reduction interventions (19). Our analysis suggests that removing social and systematic barriers to safe and non-judgmental abortion care may reduce the predominant experiences of isolation, violence, and secrecy that impact psychosocial outcomes and overall health of AGYW (25). The admixture of socially imposed and internalized stigma and self-learned resilience of AGYW in post-abortion settings in Africa depict the complexity of psychosocial experiences in accessing reproductive healthcare. To improve the sexual and reproductive health outcomes of AGYW and prevent mortality associated with unsafe and clandestine abortion globally, it is necessary to broaden AGYW's access to psychological and sexual health services, provide advocacy and support for AGYW within communities, and build upon the empowerment that some AGYW experience.

## Data Availability Statement

The original contributions presented in the study are included in the article/Supplementary Material, further inquiries can be directed to the corresponding author/s.

## Author Contributions

YZ and RH developed and registered the systematic review protocol and conducted the review, synthesized data, interpreted results, and drafted the manuscript. NM, KN, JO, EB, EC, and EA contributed to the results interpretation and edited the manuscript. All authors approved the final version of the manuscript.

## Conflict of Interest

The authors declare that the research was conducted in the absence of any commercial or financial relationships that could be construed as a potential conflict of interest.
