# Supplementary material for: Psychosocial Experiences of Adolescent Girls and Young Women Subsequent to an Abortion in Sub-saharan Africa and Globally: A Systematic Review
Source: Front Reprod Health. 2021 May 19;3:638013. doi: 10.3389/frph.2021.638013 (PMC9580653; doi:10.3389/frph.2021.638013)
Supplement: Supplementary file 1 [file Data_Sheet_1.docx]

**PubMed**

|  | **Concept: abortion** | **Concept: mental health** | **Concept: young women** |
| --- | --- | --- | --- |
| Subject Headings | Abortion, Induced[mh] | mental health[mh]  mental disorders[mh]  mood disorders[mh]  anxiety disorders[mh]  Anxiety[mh]  Depression[mh]  Patient Health Questionnaire[mh]  Abortion, Induced/psychology[mh]  Stress Disorders, Post-Traumatic[mh]  Quality of Life[mh]  Adjustment Disorders[mh] | Adolescent[mh]  Young adult[mh] |
| Free text terms  (searched in title & abstract [tiab], and in author keywords [ot]) | abortion  “pregnancy termination”  “termination of pregnancy”  **“**pregnancy interruption**”**  **“dilation and evacuation”**  **“vacuum aspiration”** | mental health  mental disorders  mental disorder  psychological disorders  psychological disorder  mood disorders  mood disorder  anxiety disorders  anxiety disorder  anxiety  Depression  depressive  Patient Health Questionnaire  phq 9  psychosocial adjustments  psychosocial adjustment  psychosocial outcomes  psychosocial outcome  post-traumatic stress disorder  posttraumatic stress disorder  quality of life  adjustment disorders  adjustment disorder  Stigma | Adolescent  Adolescents  Young adult  Young adults  young woman  young women  Girl  Girls  Teen  Teens  Youth  Youths |

(Abortion, Induced[mh] OR abortion[tiab] OR “pregnancy termination”[tiab] OR “termination of pregnancy”[tiab] OR **“**pregnancy interruption**”[tiab] OR “dilation and evacuation”[tiab] OR “vacuum aspiration”[tiab]** OR abortion[ot] OR “pregnancy termination”[ot] OR “termination of pregnancy”[ot] OR **“**pregnancy interruption**”[ot] OR “dilation and evacuation”[ot] OR “vacuum aspiration”[ot]**)

AND

(mental health[MeSH Terms] OR mental disorders[MeSH Terms] OR mood disorders[mh] OR anxiety disorders[mh] OR Anxiety[mh] OR Depression[mh] OR Patient Health Questionnaire[mh] OR Abortion, Induced/psychology[mh] OR Stress Disorders, Post-Traumatic[mh] OR Quality of Life[mh] OR Adjustment Disorders[mh] OR mental health[tiab] OR mental disorders[tiab] OR mental disorder[tiab] OR psychological disorders[tiab] OR psychological disorder[tiab] OR mood disorders[tiab] OR mood disorder[tiab] OR anxiety disorders[tiab] OR anxiety disorder[tiab] OR anxiety[tiab] OR Depression[tiab] OR depressive[tiab] OR Patient Health Questionnaire[tiab] OR phq 9[tiab] OR psychosocial adjustments[tiab] OR psychosocial adjustment[tiab] OR psychosocial outcomes[tiab] OR psychosocial outcome[tiab] OR post-traumatic stress disorder[tiab] OR posttraumatic stress disorder[tiab] OR quality of life[tiab] OR adjustment disorders[tiab] OR adjustment disorder[tiab] OR Stigma[tiab] OR mental health[ot] OR mental disorders[ot] OR mental disorder[ot] OR psychological disorders[ot] OR psychological disorder[ot] OR mood disorders[ot] OR mood disorder[ot] OR anxiety disorders[ot] OR anxiety disorder[ot] OR anxiety[ot] OR Depression[ot] OR depressive[ot] OR Patient Health Questionnaire[ot] OR phq 9[ot] OR psychosocial adjustments[ot] OR psychosocial adjustment[ot] OR psychosocial outcomes[ot] OR psychosocial outcome[ot] OR post-traumatic stress disorder[ot] OR posttraumatic stress disorder[ot] OR quality of life[ot] OR adjustment disorders[ot] OR adjustment disorder[ot] OR Stigma[ot])

AND

(Adolescent[mh] OR Young adult[mh] OR Adolescent[tiab] OR Adolescents[tiab] OR Young adult[tiab] OR Young adults[tiab] OR young woman[tiab] OR young women[tiab] OR Girl[tiab] OR Girls[tiab] OR Teen[tiab] OR Teens[tiab] OR Youth[tiab] OR Youths[tiab] OR Adolescent[ot] OR Adolescents[ot] OR Young adult[ot] OR Young adults[ot] OR young woman[ot] OR young women[ot] OR Girl[ot] OR Girls[ot] OR Teen[ot] OR Teens[ot] OR Youth[ot] OR Youths[ot])

Results: 1,648

**Embase**

|  | **Concept: abortion** | **Concept: mental health** | **Concept: young women** |
| --- | --- | --- | --- |
| Subject Headings | 'pregnancy termination'/exp  ‘elective abortion'/exp | 'mental health'/exp  'mental disease'/exp  ‘adjustment disorders’/exp  ‘anxiety disorders’/exp  'posttraumatic stress disorder'/exp  ‘mood disorders’/exp  ‘anxiety’/de  ‘depression’/exp  'distress syndrome'/exp  'psychological aspect'/exp  **‘Quality of Life’/exp**  **'patient health questionnaire'/exp**  ‘stigma’/exp | 'adolescent'/exp  'young adult'/exp  ‘girl’/exp |
| Free text terms  (searched in title, abstract, and keyword (:ti,ab,kw)) | Pregnancy-termination  Termination-of-pregnancy  Induced-abortion  elective-abortion*  abortion-induction  artificial-abortion  pregnancy-interruption  provoked-abortion  dilation-and-evacuation  illegal-abortion  legal-abortion  medical-abortion  selective-abortion  surgical-abortion  therapeutic-abortion  vacuum-aspiration | **mental-health**  **mental-disorder***  psychological-distress  **psychological-disorder***  **mood-disorder***  **anxiety-disorder***  **anxiety**  **Depression**  **depressive**  **Patient-Health-Questionnaire**  **phq-9**  **psychosocial-adjustment***  **psychosocial-outcome***  **post-traumatic-stress-disorder***  **posttraumatic-stress-disorder***  distress-syndrome  psychological-aspect  **quality-of-life**  **adjustment-disorder***  stigma | Adolescent*  Young-adult*  young-woman  young-women  Girl*  Teen*  Youth* |

('pregnancy termination'/exp OR ‘elective abortion'/exp OR Pregnancy-termination:ti,ab,kw OR Termination-of-pregnancy:ti,ab,kw OR Induced-abortion:ti,ab,kw OR elective-abortion*:ti,ab,kw OR abortion-induction:ti,ab,kw OR artificial-abortion:ti,ab,kw OR pregnancy-interruption:ti,ab,kw OR provoked-abortion:ti,ab,kw OR dilation-and-evacuation:ti,ab,kw OR illegal-abortion:ti,ab,kw OR legal-abortion:ti,ab,kw OR medical-abortion:ti,ab,kw OR selective-abortion:ti,ab,kw OR surgical-abortion:ti,ab,kw OR therapeutic-abortion:ti,ab,kw OR vacuum-aspiration:ti,ab,kw)

AND

('mental health'/exp OR 'mental disease'/exp OR ‘adjustment disorders’/exp OR ‘anxiety disorders’/exp OR 'posttraumatic stress disorder'/exp OR ‘mood disorders’/exp OR ‘anxiety’/de OR ‘depression’/exp OR 'distress syndrome'/exp OR 'psychological aspect'/exp OR **‘Quality of Life’/exp OR 'patient health questionnaire'/exp OR** ‘stigma’/exp OR **mental-health:ti,ab,kw OR mental-disorder*:ti,ab,kw OR** psychological-distress**:ti,ab,kw** OR **psychological-disorder*:ti,ab,kw OR mood-disorder*:ti,ab,kw OR anxiety-disorder*:ti,ab,kw OR anxiety:ti,ab,kw OR Depression:ti,ab,kw OR depressive:ti,ab,kw OR Patient-Health-Questionnaire:ti,ab,kw OR phq-9:ti,ab,kw OR psychosocial-adjustment*:ti,ab,kw OR psychosocial-outcome*:ti,ab,kw OR post-traumatic-stress-disorder*:ti,ab,kw OR posttraumatic-stress-disorder*:ti,ab,kw OR** distress-syndrome:ti,ab,kw OR psychological-aspect:ti,ab,kw OR **quality-of-life:ti,ab,kw OR adjustment-disorder*:ti,ab,kw OR** stigma:ti,ab,kw)

AND

('adolescent'/exp OR 'young adult'/exp OR ‘girl’/exp OR Adolescent*:ti,ab,kw OR Young-adult*:ti,ab,kw OR young-woman:ti,ab,kw OR young-women:ti,ab,kw OR Girl*:ti,ab,kw OR Teen*:ti,ab,kw OR Youth*:ti,ab,kw)

Results: 1,131; 158 exclusive to Embase

**PsycInfo**

|  | **Concept: abortion** | **Concept: mental health** | **Concept: young women** |
| --- | --- | --- | --- |
| PsycInfo Subject Headings | DE "Induced Abortion" | DE "Mental Health"  DE "Mental Disorders"  DE "Affective Disorders"  DE "Major Depression"  DE "Anaclitic Depression"  DE "Dysthymic Disorder"  DE "Endogenous Depression"  DE "Late Life Depression"  DE "Postpartum Depression"  DE "Reactive Depression"  DE "Recurrent Depression"  DE "Treatment Resistant Depression"  DE "Anxiety Disorders"  DE "Generalized Anxiety Disorder"  DE "Obsessive Compulsive Disorder"  DE "Panic Attack"  DE "Panic Disorder"  DE "Phobias"  DE "Separation Anxiety Disorder"  DE "Trichotillomania"  DE "Bipolar Disorder"  DE "Borderline States"  DE "Chronic Mental Illness"  DE "Dissociative Disorders"  DE "Eating Disorders"  DE "Gender Dysphoria"  DE "Mental Disorders due to General Medical Conditions"  DE "Neurocognitive Disorders"  DE "Neurodevelopmental Disorders"  DE "Neurosis"  DE "Paraphilias"  DE "Personality Disorders"  DE "Psychosis"  DE "Serious Mental Illness"  DE "Sleep Wake Disorders"  DE "Somatoform Disorders"  DE "Stress and Trauma Related Disorders"  DE "Acute Stress Disorder"  DE "Adjustment Disorders"  DE "Attachment Disorders"  DE "Posttraumatic Stress Disorder"  DE "Complex PTSD"  DE "DESNOS"  DE "Substance Related and Addictive Disorders"  DE "Thought Disturbances"  DE "Anxiety"  DE "Quality of Life"  DE "Self-Stigma"  DE "Stigma"  DE "Psychosocial Outcomes" | DE "Emerging Adulthood"  DE "Adolescent Pregnancy"  DE "Adolescent Mothers" |
| MeSH terms (MA) | Abortion, Induced | “mental health”  “mental disorders”  “mood disorders”  “anxiety disorders”  “Anxiety”  “Depression”  “Abortion, Induced/psychology”  “Stress Disorders, Post-Traumatic”  “Quality of Life”  “Adjustment Disorders”  “Patient Health Questionnaire” | Adolescent  "Young adult" |
| Free text terms  (searched in Title, Abstract, and Keywords) | “Pregnancy termination” OR “Termination of pregnancy” OR “Induced abortion” OR “elective abortion” OR “abortion induction” OR “artificial abortion” OR “pregnancy interruption” OR “provoked abortion” OR “dilation and evacuation” OR “illegal abortion” OR “legal abortion” OR “medical abortion” OR “selective abortion” OR “surgical abortion” OR “therapeutic abortion” OR “vacuum aspiration” | “mental health”  “mental disorder”  “mental disorders”  “psychological distress”  “psychological disorder”  “psychological disorders”  “mood disorder”  “mood disorders”  “adjustment disorder”  “adjustment disorders”  “anxiety disorder”  “anxiety disorders”  “anxiety”  “Depression”  “depressive”  “Patient Health Questionnaire”  “phq 9”  “psychosocial adjustment”  “psychosocial adjustments”  “psychosocial outcome”  “psychosocial outcomes”  “post traumatic stress disorder”  “post traumatic stress disorders”  “posttraumatic stress disorder”  “posttraumatic stress disorders”  “quality of life”  “stigma” | “Adolescent”  “Adolescents”  “Young adult”  “Young adults”  “young woman”  “young women”  “Girl”  “Girls”  “Teen”  “Teens”  “Youth”  “Youths” ) |

**With age groups in string:**

(DE "Induced Abortion" OR MA Abortion, Induced OR TI ( “Pregnancy termination” OR “Termination of pregnancy” OR “Induced abortion” OR “elective abortion” OR “abortion induction” OR “artificial abortion” OR “pregnancy interruption” OR “provoked abortion” OR “dilation and evacuation” OR “illegal abortion” OR “legal abortion” OR “medical abortion” OR “selective abortion” OR “surgical abortion” OR “therapeutic abortion” OR “vacuum aspiration” ) OR AB ( “Pregnancy termination” OR “Termination of pregnancy” OR “Induced abortion” OR “elective abortion” OR “abortion induction” OR “artificial abortion” OR “pregnancy interruption” OR “provoked abortion” OR “dilation and evacuation” OR “illegal abortion” OR “legal abortion” OR “medical abortion” OR “selective abortion” OR “surgical abortion” OR “therapeutic abortion” OR “vacuum aspiration” ) OR KW ( “Pregnancy termination” OR “Termination of pregnancy” OR “Induced abortion” OR “elective abortion” OR “abortion induction” OR “artificial abortion” OR “pregnancy interruption” OR “provoked abortion” OR “dilation and evacuation” OR “illegal abortion” OR “legal abortion” OR “medical abortion” OR “selective abortion” OR “surgical abortion” OR “therapeutic abortion” OR “vacuum aspiration” ))

AND

(( DE "Mental Health" OR DE "Mental Disorders" OR DE "Affective Disorders" OR DE "Major Depression" OR DE "Anaclitic Depression" OR DE "Dysthymic Disorder" OR DE "Endogenous Depression" OR DE "Late Life Depression" OR DE "Postpartum Depression" OR DE "Reactive Depression" OR DE "Recurrent Depression" OR DE "Treatment Resistant Depression" OR DE "Anxiety Disorders" OR DE "Generalized Anxiety Disorder" OR DE "Obsessive Compulsive Disorder" OR DE "Panic Attack" OR DE "Panic Disorder" OR DE "Phobias" OR DE "Separation Anxiety Disorder" OR DE "Trichotillomania" OR DE "Bipolar Disorder" OR DE "Borderline States" OR DE "Chronic Mental Illness" OR DE "Dissociative Disorders" OR DE "Eating Disorders" OR DE "Gender Dysphoria" OR DE "Mental Disorders due to General Medical Conditions" OR DE "Neurocognitive Disorders" OR DE "Neurodevelopmental Disorders" OR DE "Neurosis" OR DE "Paraphilias" OR DE "Personality Disorders" OR DE "Psychosis" OR DE "Serious Mental Illness" OR DE "Sleep Wake Disorders" OR DE "Somatoform Disorders" OR DE "Stress and Trauma Related Disorders" OR DE "Acute Stress Disorder" OR DE "Adjustment Disorders" OR DE "Attachment Disorders" OR DE "Posttraumatic Stress Disorder" OR DE "Complex PTSD" OR DE "DESNOS" OR DE "Substance Related and Addictive Disorders" OR DE "Thought Disturbances" OR DE "Anxiety" OR DE "Quality of Life" OR DE "Self-Stigma" OR DE "Stigma" OR DE "Psychosocial Outcomes" ) OR MA ( “mental health” OR “mental disorders” OR “mood disorders” OR “anxiety disorders” OR “Anxiety” OR “Depression” OR “Abortion, Induced/psychology” OR “Stress Disorders, Post-Traumatic” OR “Quality of Life” OR “Adjustment Disorders” OR “Patient Health Questionnaire” ) OR TI ( “mental health” OR “mental disorder” OR “mental disorders” OR “psychological distress” OR “psychological disorder” OR “psychological disorders” OR “mood disorder” OR “mood disorders” OR “adjustment disorder” OR “adjustment disorders” OR “anxiety disorder” OR “anxiety disorders” OR “anxiety” OR “Depression” OR “depressive” OR “Patient Health Questionnaire” OR “phq 9” OR “psychosocial adjustment” OR “psychosocial adjustments” OR “psychosocial outcome” OR “psychosocial outcomes” OR “post traumatic stress disorder” OR “post traumatic stress disorders” OR “posttraumatic stress disorder” OR “posttraumatic stress disorders” OR “quality of life” OR “stigma” ) OR AB ( “mental health” OR “mental disorder” OR “mental disorders” OR “psychological distress” OR “psychological disorder” OR “psychological disorders” OR “mood disorder” OR “mood disorders” OR “adjustment disorder” OR “adjustment disorders” OR “anxiety disorder” OR “anxiety disorders” OR “anxiety” OR “Depression” OR “depressive” OR “Patient Health Questionnaire” OR “phq 9” OR “psychosocial adjustment” OR “psychosocial adjustments” OR “psychosocial outcome” OR “psychosocial outcomes” OR “post traumatic stress disorder” OR “post traumatic stress disorders” OR “posttraumatic stress disorder” OR “posttraumatic stress disorders” OR “quality of life” OR “stigma” ) OR KW ( “mental health” OR “mental disorder” OR “mental disorders” OR “psychological distress” OR “psychological disorder” OR “psychological disorders” OR “mood disorder” OR “mood disorders” OR “adjustment disorder” OR “adjustment disorders” OR “anxiety disorder” OR “anxiety disorders” OR “anxiety” OR “Depression” OR “depressive” OR “Patient Health Questionnaire” OR “phq 9” OR “psychosocial adjustment” OR “psychosocial adjustments” OR “psychosocial outcome” OR “psychosocial outcomes” OR “post traumatic stress disorder” OR “post traumatic stress disorders” OR “posttraumatic stress disorder” OR “posttraumatic stress disorders” OR “quality of life” OR “stigma” ))

AND

(( DE "Emerging Adulthood" OR DE "Adolescent Pregnancy" OR DE "Adolescent Mothers" ) OR MA ( Adolescent OR "Young adult" ) OR TI ( “Adolescent” OR “Adolescents” OR “Young adult” OR “Young adults” OR “young woman” OR “young women” OR “Girl” OR “Girls” OR “Teen” OR “Teens” OR “Youth” OR “Youths” ) OR AB ( “Adolescent” OR “Adolescents” OR “Young adult” OR “Young adults” OR “young woman” OR “young women” OR “Girl” OR “Girls” OR “Teen” OR “Teens” OR “Youth” OR “Youths” ) OR KW ( “Adolescent” OR “Adolescents” OR “Young adult” OR “Young adults” OR “young woman” OR “young women” OR “Girl” OR “Girls” OR “Teen” OR “Teens” OR “Youth” OR “Youths” ))

Results: 221

**Cumulative Index to Nursing and Allied Health Literature (CINAHL)**

|  | **Concept: abortion** | **Concept: mental health** | **Concept: young women** |
| --- | --- | --- | --- |
| Subject Headings | (MH "Abortion, Induced")  (MH "Abortion, Criminal") | (MH "Mental Health")  (MH "Mental Disorders+")  (MH "Adjustment Disorders+")  (MH "Neurotic Disorders+")  (MH "Affective Disorders+")  (MH "Depression+")  (MH "Anxiety Disorders+")  (MH "Stress Disorders, Post-Traumatic")  (MH "Anxiety+")  (MH "Psychological Distress")  (MH "Quality of Life")  (MH "Stigma")  (MH "Abortion, Induced/PF") | (MH "Adolescence+")  (MH "Adolescent Parents+")  (MH "Pregnancy in Adolescence+")  (MH "Maternal Age 14 and Under")  (MH "Young Adult") |
| Free text terms  (searched in Title & Abstract; keyword not a searchable field) | “Pregnancy termination”  “Termination of pregnancy”  “Induced abortion”  “elective abortion”  “abortion induction”  “artificial abortion”  “pregnancy interruption”  “provoked abortion”  “dilation and evacuation”  “illegal abortion”  “legal abortion”  “medical abortion”  “selective abortion”  “surgical abortion”  “therapeutic abortion”  “vacuum aspiration” | “mental health”  “mental disorders”  “mental disorder”  “psychological disorders”  “psychological disorder”  “mood disorders”  “mood disorder”  “anxiety disorders”  “anxiety disorder”  “anxiety”  “Depression”  “depressive”  “Patient Health Questionnaire”  “phq 9”  “psychosocial adjustments”  “psychosocial adjustment”  “psychosocial outcomes”  “psychosocial outcome”  “post-traumatic stress disorder”  “posttraumatic stress disorder”  “quality of life”  “adjustment disorders”  “adjustment disorder”  “Stigma” | “Adolescent”  “Adolescents”  “Young adult”  “Young adults”  “young woman”  “young women”  “Girl”  “Girls”  “Teen”  “Teens”  “Youth”  “Youths” |

(( (MH "Abortion, Induced") OR (MH "Abortion, Criminal") ) OR TI ( “Pregnancy termination” OR “Termination of pregnancy” OR “Induced abortion” OR “elective abortion” OR “abortion induction” OR “artificial abortion” OR “pregnancy interruption” OR “provoked abortion” OR “dilation and evacuation” OR “illegal abortion” OR “legal abortion” OR “medical abortion” OR “selective abortion” OR “surgical abortion” OR “therapeutic abortion” OR “vacuum aspiration” ) OR AB ( “Pregnancy termination” OR “Termination of pregnancy” OR “Induced abortion” OR “elective abortion” OR “abortion induction” OR “artificial abortion” OR “pregnancy interruption” OR “provoked abortion” OR “dilation and evacuation” OR “illegal abortion” OR “legal abortion” OR “medical abortion” OR “selective abortion” OR “surgical abortion” OR “therapeutic abortion” OR “vacuum aspiration” ))

AND

(( (MH "Mental Health") OR (MH "Mental Disorders+") OR (MH "Adjustment Disorders+") OR (MH "Neurotic Disorders+") OR (MH "Affective Disorders+") OR (MH "Depression+") OR (MH "Anxiety Disorders+") OR (MH "Stress Disorders, Post-Traumatic") OR (MH "Anxiety+") OR (MH "Psychological Distress") OR (MH "Quality of Life") OR (MH "Stigma") OR (MH "Abortion, Induced/PF") ) OR TI ( “mental health” OR “mental disorders” OR “mental disorder” OR “psychological disorders” OR “psychological disorder” OR “mood disorders” OR “mood disorder” OR “anxiety disorders” OR “anxiety disorder” OR “anxiety” OR “Depression” OR “depressive” OR “Patient Health Questionnaire” OR “phq 9” OR “psychosocial adjustments” OR “psychosocial adjustment” OR “psychosocial outcomes” OR “psychosocial outcome” OR “post-traumatic stress disorder” OR “posttraumatic stress disorder” OR “quality of life” OR “adjustment disorders” OR “adjustment disorder” OR “Stigma” ) OR AB ( “mental health” OR “mental disorders” OR “mental disorder” OR “psychological disorders” OR “psychological disorder” OR “mood disorders” OR “mood disorder” OR “anxiety disorders” OR “anxiety disorder” OR “anxiety” OR “Depression” OR “depressive” OR “Patient Health Questionnaire” OR “phq 9” OR “psychosocial adjustments” OR “psychosocial adjustment” OR “psychosocial outcomes” OR “psychosocial outcome” OR “post-traumatic stress disorder” OR “posttraumatic stress disorder” OR “quality of life” OR “adjustment disorders” OR “adjustment disorder” OR “Stigma” ))

AND

(( (MH "Adolescence+") OR (MH "Adolescent Parents+") OR (MH "Pregnancy in Adolescence+") OR (MH "Maternal Age 14 and Under") OR (MH "Young Adult") ) OR TI ( “Adolescent” OR “Adolescents” OR “Young adult” OR “Young adults” OR “young woman” OR “young women” OR “Girl” OR “Girls” OR “Teen” OR “Teens” OR “Youth” OR “Youths” ) OR AB ( “Adolescent” OR “Adolescents” OR “Young adult” OR “Young adults” OR “young woman” OR “young women” OR “Girl” OR “Girls” OR “Teen” OR “Teens” OR “Youth” OR “Youths” ))

Results: 549
